# *KCC2* expression levels are reduced in post mortem brain tissue of Rett syndrome patients

**DOI:** 10.1186/s40478-019-0852-x

**Published:** 2019-12-03

**Authors:** Lisa Hinz, Joan Torrella Barrufet, Vivi M. Heine

**Affiliations:** 1 Department of Complex Trait Genetics, Center for Neurogenomics and Cognitive Research, Amsterdam Neuroscience, Vrije Universiteit, Amsterdam, The Netherlands; 2 Pediatric Neurology, Emma Children’s Hospital, Amsterdam UMC, Vrije Universiteit Amsterdam, Amsterdam Neuroscience, Boelelaan 1085, 1081HV Amsterdam, The Netherlands

**Keywords:** Rett syndrome, KCC2-deficiency, E/I imbalance, Neuronal network immaturity

## Abstract

Rett Syndrome (RTT) is a neurodevelopmental disorder caused by mutations in the Methyl CpG binding protein 2 (*MECP2*) gene. Deficient K^+^-Cl^—^co-transporter 2 (*KCC2*) expression is suggested to play a key role in the neurodevelopmental delay in RTT patients’ neuronal networks. KCC2 is a major player in neuronal maturation by supporting the GABAergic switch, through the regulation of neuronal chlorine homeostasis. Previous studies suggest that MeCP2 mutations lead to changed *KCC2* expression levels, thereby causing a disturbance in excitation/inhibition (E/I) balance. To investigate this, we performed protein and RNA expression analysis on post mortem brain tissue from RTT patients and healthy controls. We showed that *KCC2* expression, in particular the *KCC2a* isoform, is relatively decreased in RTT patients. The expression of Na^+^-K^+^-Cl^−^ co-transporter 1 (*NKCC1*), responsible for the inward transport of chlorine, is not affected, leading to a reduced *KCC2*/*NKCC1* ratio in RTT brains. Our report confirms *KCC2* expression alterations in RTT patients in human brain tissue, which is in line with other studies, suggesting affected E/I balance could underlie neurodevelopmental defects in RTT patients.

## Introduction

Rett syndrome (RTT) is a progressive neurodevelopmental disorder mainly affecting young girls. First clinical symptoms typically appear between 6 and 18 months of age, involving mental impairment, stereotypic behaviour, breathing abnormalities and severe seizures [3, 4, 18, 25]. Pathological studies showed that patients with RTT have a neuronal maturation defect with smaller soma, shorter dendrites, reduced synapses and reduced inhibition, leading to severe seizures [4, 18, 25]. Classical RTT is caused by various mutations within the Methyl-CpG-binding-protein 2 gene (*MECP2*), which protein has key functions in gene regulation and chromatin modulation. Because of its DNA-binding properties, lack of functional MeCP2 is affecting hundreds of target genes, complicating the insight into how MeCP2 deficiencies lead to brain abnormalities in RTT. One of the genes that is thought to play a critical role in delayed neuronal development in Rett patients, is the K^+^-Cl^−^ co-transporter 2 (KCC2).

KCC2 is a chlorine transporter that regulates the intracellular chloride homeostasis in neurons together with the Na^+^-K^+^-Cl^−^ co-transporter 1 (NKCC1) [6]. During early brain development, NKCC1 is the dominant transporter, thereby keeping the intracellular chloride concentration high. As gamma-aminobutyric acid (GABA) transmission is connected to the chloride gradient, GABA has excitatory functions in young neurons [8]. KCC2 exists in two isoforms, KCC2a and KCC2b [30]. During neuronal maturation, KCC2b levels in particular gradually increase, while KCC2a levels only moderately change [31]. Increase in KCC2, results in lower intracellular chloride concentrations and a reversal of the chloride gradient. Consequently, the function of GABA in neurotransmission switches from excitation to inhibition [7, 9, 13, 19].

KCC2 level deficits are found in both rodent and human induced pluripotent stem cell models for RTT [5, 14, 18, 27]. Interestingly, KCC2-deficient mice show similarities to *Mecp2*-knockout mice, including poor motor abilities, breathing abnormalities, impaired learning abilities and severe seizures [5, 17, 21, 23, 28, 29]. Based on excitatory neurotransmission, *Kcc2*-knockout mice display an immature neuronal phenotype and *Mecp2*-knockouts show a reduced GABAergic inhibition. In iPSC-based models, it has been shown that overexpression of KCC2 in iPSC-derived neurons from RTT patients can rescue GABAergic function [27], and it has been hypothesized that changes in neuronal development are linked to affected KCC2 levels.

In RTT patients, researchers have previously reported a decrease in KCC2 protein level in cerebrospinal fluid (CSF) samples of 2 to 29 year old female RTT patients and an altered KCC2/NKCC1 ratio when compared to control samples [11]. More recently, these findings were confirmed on motor cortex and cerebellar tissue from RTT patients describing a reduction in *KCC2* expression levels [15]. However, the effect on *KCC2* levels in other brain regions of RTT patients was not investigated. Here we investigated *KCC2* levels and *KCC2/NKCC1* ratio in post mortem tissue of patients and controls in the areas Brodmann Area (BA) 4, BA6, BA10, BA20 and Hippocampus, using RNA and immunocytochemical analysis. We found that *KCC2* expression is overall decreased in RTT patient post mortem tissue, suggesting playing a role in neuronal development. In particular, we identified *KCC2a* but not *KCC2b* expression being significantly decreased. Our findings, together with previous studies suggest, that alteration of KCC2 levels in RTT patients play a role in disease progression and support the hypothesis of ion channel gene dysfunction in RTT.

## Case report

### Reduction of KCC2 immunofluorescence in post mortem brain tissue from RTT patients

In this case report, we compared post mortem brain tissue from three female RTT patients with an average age of 20 years (#UMB 4516, 20,98 years, p.R255X mutation in *MECP2*; #UMB 4882, 17.85 years, p.R255X mutation in *MECP2*; #UMB 5723, 22.5 years, no information about mutation available) with three gender and age matched controls (#UMB 5602, 22.14 years; #UMB 5646, 20.45 years; #UMB 5670, 17.27 years). Samples from different BA were requested (BA4, BA6, BA10, BA20 and hippocampus) and kindly provided by the NICHD Brain and Tissue Bank for Developmental Disorders, University of Maryland, Baltimore, USA. Samples were shipped as deep-frozen tissue blocks and stored at − 80 °C upon arrival.

First, we set out to confirm previous findings of reduced KCC2 levels in human CSF and mouse models, by performing immunocytochemistry for KCC2 and MeCP2 on BA20 of the provided patient and control material. For this, 9 μm sections from BA20 were prepared on pre-cooled glass slides and fixated with 100% acetone for 10 min. Slides were blocked for 1 h and incubated with antibodies (KCC2, Neuromab, 1:500; MeCP2, Cell Signalling, 1:500) over night at 4 °C. Next day, sections were incubated with secondary antibodies (Alexa-Fluor anti rabbit 594, 1:1000, Thermo Fisher Scientific; Alexa-Fluor anti mouse 488, 1:1000, Thermo Fisher Scientific) for 1 h at RT, followed by DAPI treatment for 5 min. Slides were mounted with Fluoromount G (Thermo Fisher Scientific) and imaged by fluorescent microscopy (Leica). Pictures were transferred to Java-based imaging software Fiji (Analysis Find Maxima). We averaged data derived from 2 images per patient or healthy control.

**Fig. 1:**
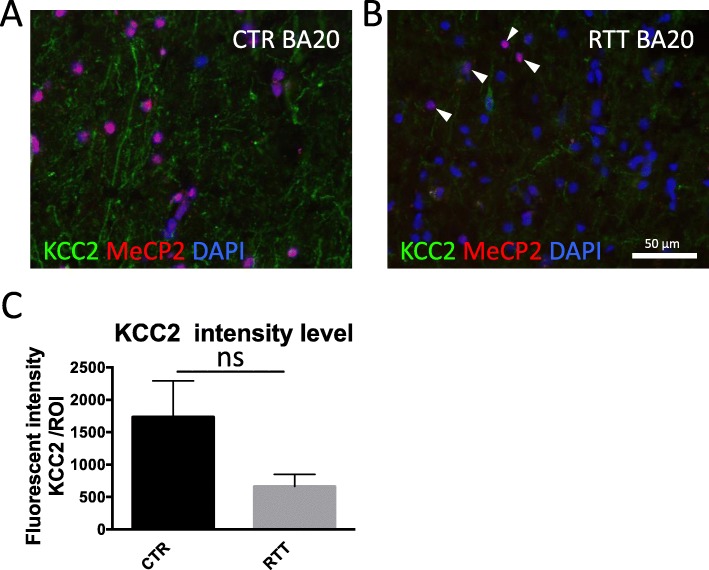
KCC2 immunohistochemistry in RTT patient brain. Representative image of immunofluorescent staining for KCC2 and MeCP2 in BA 20 of control (**a**) and RTT patient (**b**) tissue. Because of mosaic expression, MeCP2-positive cells are also detected in RTT patient tissue marked by arrowheads (**b**). Fluorescent intensity measurements indicated reduced KCC2 levels in RTT patient (Mann Whitney Test, ns.) (**c**)

Sections from healthy controls presented positive MeCP2 staining in all cells (Fig. 1a), while RTT patient tissue showed a mosaicism of positive and negative nuclei (Fig. 1b). KCC2 staining was located along the dendrites and soma of neurons (Fig. 1a+b). Although, KCC2 expression levels by fluorescent intensity measurements did not reach statistical significance (Mann Whitney Test, *p* = 0.1), it was decreased in patients when compared to healthy controls (Fig. 1b+c).

### Alteration of *KCC2* RNA expression level in post mortem brain tissue of Rett syndrome patients

To further investigate if gene expression level of *KCC2* is altered in samples of RTT patients’ brain tissue, we analysed RNA levels by quantitative real time polymerase chain reaction (qPCR). Therefore, RNA was collected from frozen post mortem brain tissue, using standardized trizol/chloroform-RNA isolation followed by reverse transcriptase PCR with SuperScript IV Reverse Transcriptase (Thermo Fisher Scientific). For qPCR analysis, SYBR Green Real-Time PCR Master Mix was used with 100 ng of sample cDNA as well as forward and reverse primer of required gene of interest (Table 1). qPCR analysis was performed with Roche Light Cycler 480 and results were analysed by using Roche Light Cycler 480 Software.

**Table 1:** Primers qPCR

1	*RPL13* forward	GAGACAGTTCTGCTGAAGAACTGAA
2	*RPL13* reverse	TCCGGACGGGCATGAC
3	*GAPDH* forward	TCAAGGGCATCCTGGGCTAC
4	*GAPDH* reverse	CGTCAAAGGTGGAGGAGTGG
5	*KCC2* forward	ACATCTTTGGCGTCATCCTC
6	*KCC2* reverse	CAGGCACAACACCATTCGTT
7	*NKCC1* forward	CCGATTTTCGAGAGGAAGAG
8	*NKCC1* reverse	TGCAATTCCTACGTAAACCAA
9	*KCC2a* forward	AGAAGCCCTGACCCAGAGTC
10	*KCC2a* reverse	CTTCTCTGTGTCGGTGCTGT
11	*KCC2b* forward	CGCCACCATGCTAAACAACC
12	*KCC2b* reverse	CTTCTCTGTGTCGGTGCTGT

**Fig. 2:**
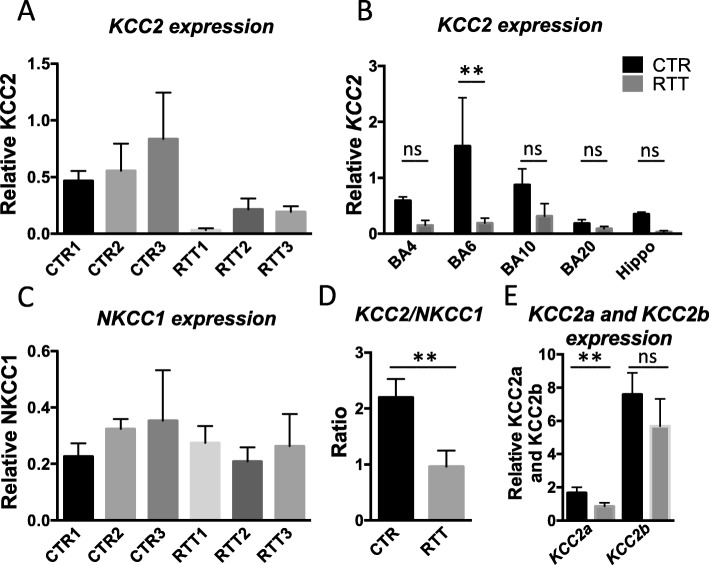
qPCR analysis for KCC2 and NKCC1 in RTT brain samples. *KCC2* expression levels are reduced in all 3 RTT patient brains compared to control 3 control brains (**a**). Reduced *KCC2* expression levels are found in all brain regions studied, but only reached statistical significance in BA6 (Multiple t test BA6 *p* < 0.01; one-way ANOVA *P* < 0,0001) (**b**). *NKCC1* expression levels are unchanged in RTT patient brains (**c**). The *KCC2/NKCC1* ratio is significantly reduced in RTT patient brains (Mann-Whitney test P < 0,007) (**d**). Averaged expression levels of *KCC2* isotypes are reduced in RTT samples, which reached statistical significance for *KCC2a* (2-Way-ANOVA *P* < 0,005) but not for *KCC2b* expression (2-Way-ANOVA, ns) (**e**)

*KCC2* levels in the RTT brain were determined by averaging the measured levels in five brain regions, BA4, BA6, BA10, BA20 and hippocampus, relative to housekeeping genes. We found that all three RTT patients studied, show a lower *KCC2* expression compared to healthy controls (Kruskal-Wallis test *p* = 0,0143, Fig. 2a). When brain regions were studied separately, *KCC2* expression levels were also decreased in patients, but only reached statistical significance in BA6 (one-way ANOVA, BA6 *p* < 0.004, BA4 n.s., BA10 n.s., BA20 n.s., Hippocampus n.s.) (Fig. 2b). No alterations were observed in *NKCC1* expression levels of RTT patients when compared to controls (Fig. 2c). Therefore, mean ratio of *KCC2* and *NKCC1* of all three patients were significantly decreased (Mann-Whitney test, *p* < 0.007) (Fig. 2d). We also looked into the expression levels of the different subtypes of *KCC2*, namely *KCC2a* and *KCC2b*. Both isoform were decreased in RTT patient samples (average of the different brain regions), but only reached statistical significance for *KCC2a* (Fig. 2e). Therefore, our results are in line with previous findings, that *KCC2* expression is affected by RTT.

## Discussion

Earlier studies investigated KCC2 level changes in RTT patient’s CSF, in human iPSC-derived neurons and in motor cortex and cerebellar tissue of RTT patients [11, 15, 27]. These findings suggest involvement of KCC2 in disease mechanisms in RTT. In support of this hypothesis and to study the influence of MeCP2 on *KCC2* levels in different brain areas, we investigated *KCC2* expression in four different cortical areas and hippocampus of three RTT patients and age matched controls. We observed a significant reduction in *KCC2* gene expression in RTT samples compared to controls. Considering KCC2 is essential for a functional E/I balance [6, 7], these insights strengthen the importance of ion channel dysfunction in RTT patient brains, as E/I imbalance are associated with delayed neuronal development and seizures in RTT.

RTT tissue was derived from three female patients. Due to random X-chromosomal inactivation during development, the derived tissue showed mosaicism for *MECP2* expression [24]. Therefore, the observed BAs did contain mixed populations of affected and unaffected neurons (Fig.1b). If we assume that MeCP2 deficiencies lead to reduced KCC2 levels, it is likely that KCC2 levels were not affected in all cells of the studied tissue. This could explain the observed decrease in fluorescent intensity of KCC2 immunostainings in RTT brain tissue without reaching statistical significance (Fig. 1c). Studying MeCP2-negative cells exclusively, could overcome this issue. But, due to pre-treatment of the brain tissue, namely freezing and cryo-sectioning, identification of individual cells was difficult. Therefore, only the average fluorescent intensity of KCC2 could be analysed.

To gain more insight into *KCC2* expression levels within RTT patient brains, we additionally performed qPCR analysis. We showed decreased *KCC2* expression in different areas of RTT patient brains compared to healthy controls (Fig. 2b). Noteworthy, we observed that *KCC2* expression was not evenly expressed over control tissue, which could reflect expression changes during developmental stages of the brain areas [22] and region-specific differences [16]. But, as controls were age-matched, these variables do not account for decreased *KCC2* levels observed. Also here mosaic expression could have played a role. However, we did observe a similar trend for all brain areas investigated, showing a reduction of *KCC2* expression in RTT compared to CTR samples. These results are in line with the immunofluorescent intensity analysis of BA20, suggesting that protein levels of KCC2 are reduced in several different brain areas in the cortex and hippocampus.

Besides *KCC2*, we also investigated *NKCC1* expression levels and did not detect expression level alterations. These findings are in line with previous studies in human CSF and brain tissue [11, 15]. However, as the KCC2/NKCC1 ratio ultimately regulates chloride homeostasis, GABA switch and maturation of the brain, changed KCC2 levels can have a major impact on brain development [6, 7, 20]. As brain tissue samples were taken from RTT patients and healthy controls in adolescence, we cannot exclude KCC2 levels normalize over time. However, by the end of adolescence, developmental processes of the brain are almost completed and KCC2 levels will most likely remain stable. Therefore, it is likely that alterations in *KCC2* expression already appear during early brain development, having a major impact on disease progression in RTT patients.

Interestingly, we identified a significant reduction in *KCC2a* but not in *KCC2b* expression levels. As *KCC2b* expression is strongly upregulated during early development, it is suggested that KCC2b is primarily responsible for the GABAergic shift [30]. However, before KCC2b levels majorly increase, KCC2a levels make up 50% of total KCC2 levels. These expression levels double in cortical areas during development, suggesting a significant role in network maturation [31]. Nonetheless, in adolescence only 8–10% of the total KCC2 levels involves KCC2a [30, 31]. Worth mentioning, an important role for KCC2a in development of respiratory ﻿rhythmogenesis has been shown, as its absence leads to abnormal breathing rates and apnoea in mice [12]. Breathing abnormalities with apnoea are common symptoms in RTT patients and are so far associated with reduced GABAergic innervation in the brain stem [1, 10, 26]. Since *KCC2a* is specifically highly expressed within the brain stem, the role of KCC2a level changes deserves further study.

Summarizing, our results confirm earlier hypotheses that RTT patient brains show a decreased *KCC2* expression, resulting in a lower *KCC2/NKCC1* ratio. Interestingly, we could identify significant changes in the expression of *KCC2a* isoform. Therefore, brains of RTT patients display an underdeveloped phenotype, which matches other phenotypic alterations, such as decreased neuronal size, reduced number of synapses and shorter dendritic outgrowth [2–4, 18, 25]. This proposes the need for sufficient treatment during these developmental processes to support brain development and reduce disease progression. By understanding these mechanisms and its alterations in RTT during early development, we can identify new treatment approaches and elucidate the need for early diagnostics.

## Data Availability

Not applicable.

## Abbreviations

BA: Brodmann Area
CSF: Cerebral Spinal Fluid
E/I: Excitation/Inhibition
GABA: Gamma-Aminobutyric Acid
KCC2: K^+^-Cl^−^ Co-Transporter 2
MeCP2: Methyl-CpG-Binding-Protein 2
NKCC1: Na^+^-K^+^-Cl^−^ Co-Transporter 1
qPCR: Quantitative Real Time Polymerase Chain Reaction
RTT: Rett Syndrome
